# A Virtual Reading Center Model Using Crowdsourcing to Grade Photographs for Trachoma: Validation Study

**DOI:** 10.2196/41233

**Published:** 2023-04-06

**Authors:** Christopher J Brady, R Chase Cockrell, Lindsay R Aldrich, Meraf A Wolle, Sheila K West

**Affiliations:** 1 Division of Ophthalmology Department of Surgery Larner College of Medicine at The University of Vermont Burlington, VT United States; 2 Division of Surgical Research Department of Surgery Larner College of Medicine at The University of Vermont Burlington, VT United States; 3 Larner College of Medicine at The University of Vermont Burlington, VT United States; 4 Dana Center for Preventive Ophthalmology Wilmer Eye Institute Baltimore, MD United States

**Keywords:** trachoma, crowdsourcing, telemedicine, ophthalmic photography, Amazon Mechanical Turk, image analysis, diagnosis, detection, cloud-based, image interpretation, disease identification, diagnostics, image grading, disease grading, trachomatous inflammation—follicular, ophthalmology

## Abstract

**Background:**

As trachoma is eliminated, skilled field graders become less adept at correctly identifying active disease (trachomatous inflammation—follicular [TF]). Deciding if trachoma has been eliminated from a district or if treatment strategies need to be continued or reinstated is of critical public health importance. Telemedicine solutions require both connectivity, which can be poor in the resource-limited regions of the world in which trachoma occurs, and accurate grading of the images.

**Objective:**

Our purpose was to develop and validate a cloud-based “virtual reading center” (VRC) model using crowdsourcing for image interpretation.

**Methods:**

The Amazon Mechanical Turk (AMT) platform was used to recruit lay graders to interpret 2299 gradable images from a prior field trial of a smartphone-based camera system. Each image received 7 grades for US $0.05 per grade in this VRC. The resultant data set was divided into training and test sets to internally validate the VRC. In the training set, crowdsourcing scores were summed, and the optimal raw score cutoff was chosen to optimize kappa agreement and the resulting prevalence of TF. The best method was then applied to the test set, and the sensitivity, specificity, kappa, and TF prevalence were calculated.

**Results:**

In this trial, over 16,000 grades were rendered in just over 60 minutes for US $1098 including AMT fees. After choosing an AMT raw score cut point to optimize kappa near the World Health Organization (WHO)–endorsed level of 0.7 (with a simulated 40% prevalence TF), crowdsourcing was 95% sensitive and 87% specific for TF in the training set with a kappa of 0.797. All 196 crowdsourced-positive images received a skilled overread to mimic a tiered reading center and specificity improved to 99%, while sensitivity remained above 78%. Kappa for the entire sample improved from 0.162 to 0.685 with overreads, and the skilled grader burden was reduced by over 80%. This tiered VRC model was then applied to the test set and produced a sensitivity of 99% and a specificity of 76% with a kappa of 0.775 in the entire set. The prevalence estimated by the VRC was 2.70% (95% CI 1.84%-3.80%) compared to the ground truth prevalence of 2.87% (95% CI 1.98%-4.01%).

**Conclusions:**

A VRC model using crowdsourcing as a first pass with skilled grading of positive images was able to identify TF rapidly and accurately in a low prevalence setting. The findings from this study support further validation of a VRC and crowdsourcing for image grading and estimation of trachoma prevalence from field-acquired images, although further prospective field testing is required to determine if diagnostic characteristics are acceptable in real-world surveys with a low prevalence of the disease.

## Introduction

### Trachoma

Despite intensive worldwide control efforts, trachoma remains the most important infectious cause of vision loss [1-3] and one of the overall leading causes of global blindness [4,5], with nearly 136 million people at risk of losing vision [6]. While the goal set by the World Health Organization (WHO) for the global elimination of trachoma as a public health problem by 2020 [7] was not met, this work contributed to dramatic reductions in the prevalence of the disease [8]. However, as with other diseases with the potential for elimination, the “last mile” may prove to be the most difficult [9,10]. With the decreased global prevalence of trachoma, there is growing recognition from the WHO and other major stakeholders that novel methods will be essential to achieve trachoma elimination [11-13].

### Crowdsourcing

Ophthalmic photography and telemedicine are well-established clinical and research tools useful for many conditions including trachoma [14-17], but the use of imaging at scale in an elimination program that operates almost exclusively in rural areas of resource-poor countries remains unknown. In addition, expert-level grading of images in a conventional telemedicine reading-center model is labor-intensive and expensive, and the high volume of images generated by multiple concurrent elimination surveys has the potential to overwhelm existing grading capacity. For these reasons, crowdsourcing using untrained citizen scientists has been proposed for large image processing tasks and has been successfully used in our prior research on other ophthalmic conditions [18-20]. Previously, our group has demonstrated that a smartphone-based imaging system can acquire high-quality everted upper eyelid photographs during a district-level trachoma survey [21]. In this report, we describe an internal validation of the “virtual reading center” (VRC) paradigm in which crowdsourcing is used to provide a first-pass grading of images to eliminate those without TF (trachomatous inflammation—follicular), thereby substantially reducing the skilled grader burden for trachoma elimination telemedicine.

## Methods

### Crowdsourcing Platform

**Figure 1:**
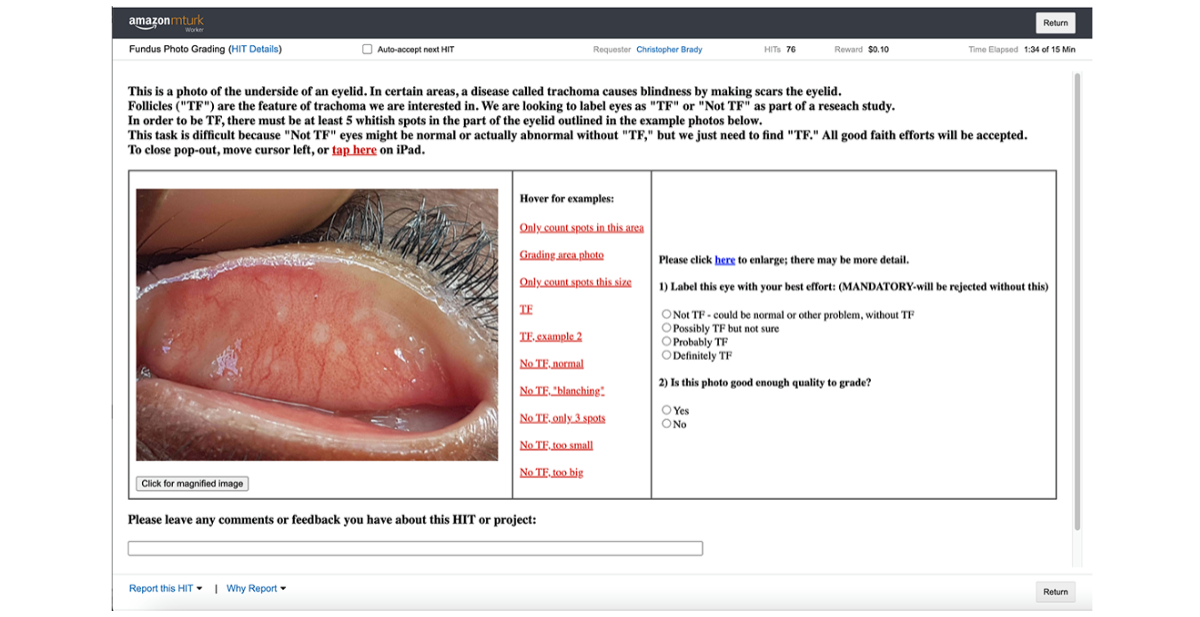
Amazon Mechanical Turk interface for active trachoma grading using crowdsourcing. HIT: human intelligence task; TF: trachomatous inflammation—follicular.

A previously designed Amazon Mechanical Turk (AMT) crowdsourcing interface for grading of retinal fundus photographs [18,20,22] was modified to allow for grading of external photographs of the everted upper eyelid (Figure 1). Annotated teaching images were included to allow AMT users to train themselves to identify the features of TF and provide their grade (0=“definitely no TF”; 1=“possibly TF”; 2=“probably TF”; 3=“definitely TF”) within the same web frame. No additional training or qualification was required before AMT users could complete the tasks. Based on previous experiments on the smallest accurate crowd size [20], 7 individual grades were requested for each image, with US $0.05 compensation provided for each grade.

### Ophthalmic Photographs

The data set of 2614 images used for this study was collected during a 2019 district-level survey in Chamwino, Tanzania, as part of the Image Capture and Processing System (ICAPS) development study [21]. During the ICAPS study, all images were assessed for gradability, operationally defined as sufficient resolution of the central upper tarsal plate to confirm or exclude the presence of 5 or more follicles by 2 experts. Gradable images then received a TF grade from 2 experts, with TF being defined as 5 or more follicles, at least 0.5 mm in diameter located in the central upper tarsal plate using the WHO simplified trachoma grading system [23]. Consensus was then decided by discussion of discordant grades. The consensus photo grade was then compared to the grade rendered by a certified field grader. All images used were completely deidentified photographs of a single everted upper eyelid (Figures S1 and S2 in Multimedia Appendix 1). The images were posted for grading on AMT in 2 batches (December 28, 2020, and March 9, 2021). For this validation study, we analyzed the set of 2299 gradable images with concordant expert and field ICAPS grades for TF (n=56 for TF-positive images). This concordant grade was considered the “ground truth” for analysis of VRC grading. Images without a concordant grade were excluded from the analysis.

### Optimizing Crowdsourcing

The images were then randomly divided into equal training and test sets. The training set was used to explore and compare several ways of processing the crowdsourced output into a binary “No TF” or “TF” score. The 7 individual AMT grader scores (0-3) were summed to create a single raw score for each image (theoretical range: 0-21). A number of dichotomization “setpoints” along this range were then compared on the basis of their accuracy with the ground truth grade.

The best fitting grading method was defined using the WHO grading criteria from the Global Trachoma Mapping Project [24] as one that could identify TF with a kappa agreement of ≥0.7 with the ground truth grade in a simulated moderate prevalence sample and produce a prevalence within ±2% of the ground truth determined prevalence in the full sample [25]. Because a VRC model was anticipated using crowdsourcing as a first pass, with skilled grader overreads for positive images, minimizing the number of false-negative images while maximizing the reduction in skilled grader burden were secondary considerations during cutoff selection.

First, to mimic the current standard of care for certification of an in-person skilled grader, a simulated intergrader agreement test (IGA) [24] was conducted using 50 randomly selected images (TF n=20 based on ICAPS concordant grade). Cohen kappa agreement with the ground truth was calculated at each raw score in the simulated IGA. The score maximizing kappa was considered as the first possible setpoint to dichotomize the crowdsourced raw scores into “No TF” and “TF” for all the images in the training set.

For the next a priori dichotomization attempt, a “naïve” or “majority rules” setpoint of 50% of the raw score was applied in the training set, in which a raw score of 11 or greater was defined as TF. Finally, the receiver operating characteristic (ROC) was then analyzed and used to explore and sequentially optimize each of the diagnostic characteristics (sensitivity, specificity, percent correct, and kappa) of various cut-offs for the binary diagnosis of TF.

Individual grader performance was also compared to the ground truth to see whether several methods of excluding unreliable scores could improve score aggregation.

The best fitting grading paradigm was then applied to the test set to evaluate the final sensitivity, specificity, percent correct, kappa agreement, and percent TF positive compared with the ground truth values.

### Ethical Considerations

This study was deemed nonhuman subjects research by the University of Vermont Institutional Review Board.

## Results

### Crowdsourcing Characteristics

The first batch of 1000 images was completed in 61 minutes, by 193 unique AMT users. The median time to grade an image was 18 (IQR 103) seconds, and the median number of images graded by each user was 15 (IQR 49) images with a mode of 1 image. The second batch of 1614 images was completed in 74 minutes, by 193 unique AMT users, with a median grading time of 16 (IQR 85) seconds. A total of 43 users worked on both batches. The median number of images graded per user was 22 (IQR 76) images, and the modal number was again only 1 image. The full distribution of images graded per user is shown in Figure S3 in Multimedia Appendix 1. The total cost for crowdsourced grading of the 2 batches, including AMT commission, was US $1098.

**Figure 2:**
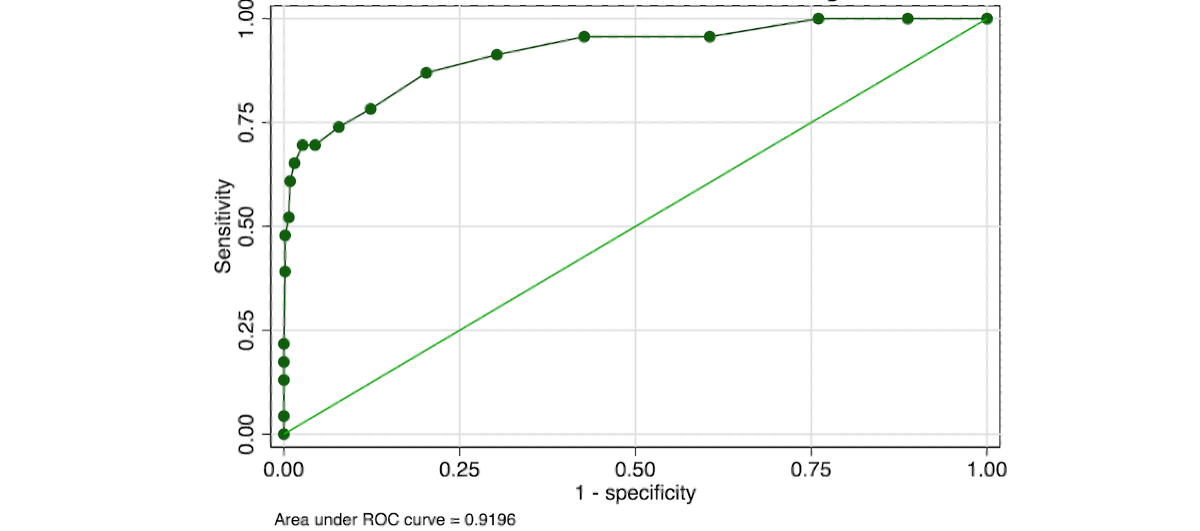
ROC of the crowdsourced raw score for the consensus ground truth grade. ROC: receiver operating characteristic.

Summing the individual scores for the training images gave a range of 0-20 with a right-skewed distribution (Figure S4 in Multimedia Appendix 1). The area under the ROC (AUROC) for the raw score was 0.920 (95% CI 0.853-0.986) (Figure 2).

### Optimizing Crowdsourcing

The results of the various attempts to optimize the aggregation of raw scores into a binary TF score using the training set of 1149 images (TF n=23; prevalence 2.00%, 95% CI 1.27%-2.99%) are shown in Table 1. Each choice of a dichotomized setpoint allows for a different balance of diagnostic parameters along the ROC. In the IGA simulation, in which the prevalence of TF was enriched to 40% (95% CI 26.4%-54.8%) in a random subset of 50 images, the optimal kappa of 0.715 and an acceptable prevalence estimate of 46% (95% CI 31.8%-60.7%) were obtained with a cutoff of 6. When the cutoff of 6 was applied in the full sample with a TF prevalence of 2.00% (95% CI 1.27%-3.00%), kappa was reduced to 0.115 and the prevalence was overestimated by a factor of 10 (estimated prevalence: 21.6%, 95% CI 19.2%-24.1%), due to the high number of false-positive results.

The next model applied used a “truncated mean” approach, in which the highest and lowest AMT individual scores for each image were excluded from the image raw score to minimize the effect of outlier scores. The range of raw scores was now 0-15, with a likewise right-skewed distribution. Once again, the IGA simulation was performed, and the maximal kappa was obtained with a lower cutoff of 4.

**Figure 3:**
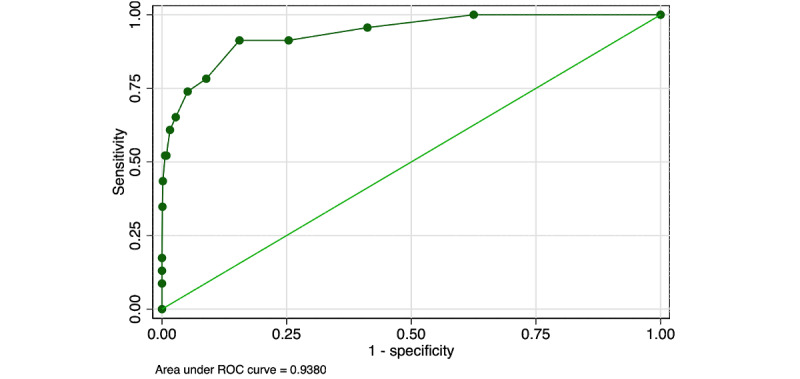
ROC with the highest and lowest score truncated from the raw score showed a slightly better area under the curve compared with the raw score. ROC: receiver operating characteristic.

Using the raw score with a truncated mean, the AUROC was 0.938 (Figure 3). Again, when the IGA-optimized cutoff was applied in the full sample, the diagnostic characteristics were reduced with a kappa of 0.162, despite a sensitivity of nearly 85% and specificity of over 90%. Because no cutoff met the prespecified criteria of kappa ≥0.7 in the IGA-simulated sample and matching prevalence of ±2% in the full sample, a VRC model was explored with skilled overreading of all positive images. Two cutoffs were explored: ≥4, which was selected from the IGA simulation with truncated means, and ≥5, which was selected as it allowed for a 90% reduction in skilled grader burden and still permitted a kappa ≥0.7 in the IGA simulation.

Using the more conservative cutoff of 4, the skilled grader was required to overread 16% of the entire data set (n=196 images), which took approximately 30 minutes using the same AMT interface. Kappa agreement with the ground truth score was 0.685, and the prevalence of TF in this sample was 2.52% (95% CI 1.7%-3.6%). Using ≥5 as a cutoff allowed for a bigger reduction in the skilled grader burden as only 10% of the images required overreading (n=115).

**Figure 4:**
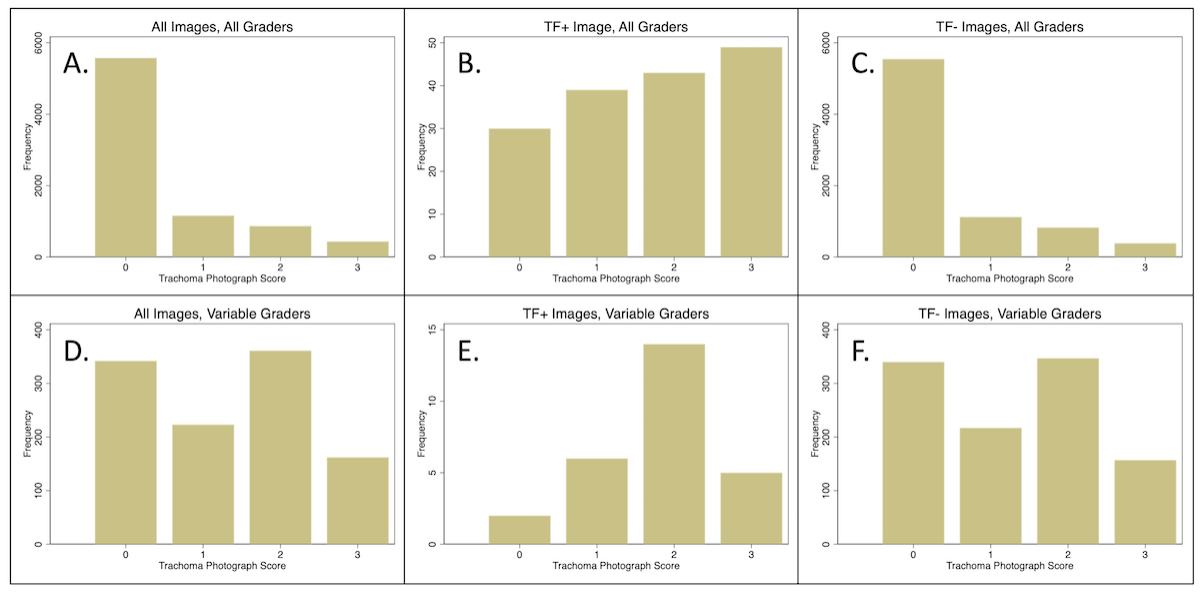
Image-level distribution of individual worker grades reveals the “fingerprint” of the entire data set (A and D) as well as images with (B and E) and without (C and F) active trachoma. In A-C, the entire set of grades is used demonstrating most images are without disease and those with disease had more scores indicating disease. In D-F, in which only less reliable grades are displayed, the difference between images with and without disease is less obvious. TF: trachomatous inflammation—follicular.

A separate set of raw score aggregation methods were attempted excluding the scores of individual AMT workers, who appeared to be less reliable. The pattern of crowdsourced responses (0-3 category usage) was reviewed at the level of each image (Figure 4A-C). Because the prevalence of TF in the data set was 2%, a reliable grader who completes a sufficient number of images should have had a median score of 0 (ie, most images should be graded as without TF). For stability, we attempted to exclude all grades from “variable” graders who had either graded fewer than 10 images (n=476 grades from 152 graders) or whose median score was >1 (n=616 grades from 14 graders; Figure 4D-F). The AUROC for this set of raw scores was 0.930 (95% CI 0.874-0.986). Neither of the cutoffs that permitted a kappa ≥0.7 in the IGA simulation generated a prevalence within ±2% of the true prevalence of the training set (data not shown).

Ultimately, the process chosen from the analysis of the training set to compute the dichotomized “No TF” versus “TF” score calculates a raw crowdsourced score after discarding the highest and lowest individual scores, designates images with truncated raw score ≥4 as preliminary positive, and then uses a skilled overread of all these preliminary positive images. We call this algorithm the “VRC method.”

For the final internal validation, the VRC method was then applied to the test set of 1150 images (TF n=33; prevalence 2.87%, 95% CI 1.98%-4.01%). In this set, 205 images required skilled overread constituting an 82% reduction in the skilled grader burden. After the overread, the kappa was 0.775, with a calculated TF prevalence of 2.70% (95% CI 1.84%-3.80%).

**Table 1:** Attempts to optimize the aggregation of raw scores using training set. In intergrader assessment (IGA) simulations, the true prevalence was 40%; otherwise, the training set gold standard prevalence was 2%.

Model and cutoff description (≥cutoff)			% correct		Sensitivity (%)		Specificity (%)		Kappa		Prevalence (%)		False positives, n		Skilled grader burden reduction (%)
**IGA simulation**															
	Maximize kappa (6)	86		90		83		0.715		46.0		N/A^a^		N/A	
**Total raw score**															
	Maximized kappa (from IGA) (6)	80		87		80		0.115		21.6		3		78	
	Naïve/majority rules (11)	98		65		98		0.534		2.8		8		97	
	WHO^b^ minimum accepted sensitivity (10)	97		70		97		0.450		4.0		7		96	
	Maximize kappa (full set) (14)	99		48		100		0.605		1.1		12		99	
	Mimic true prevalence (12)	98		61		99		0.587		2.1		9		98	
**Truncated mean approach (simulated IGA sample)**															
	Maximize kappa (4)	90		95		87		0.797		46.0		N/A		N/A	
**Truncated mean approach**															
	Maximized kappa (from IGA) (4)	85		91		84		0.162		17.1		2		84	
	Naïve/majority Rules (8)	98		61		98		0.497		2.8		9		97	
	WHO minimum accepted sensitivity (6)	94		74		95		0.326		6.5		6		93	
	Maximize kappa (full set) (11)	99		43		100		0.565		1.0		13		99	
	Mimic true prevalence (9)	98		52		99		0.524		1.9		11		98	
	Create 90% skilled grader burden reduction (5)	91		78		91		0.229		10.3		5		90	
**Virtual reading center model with overreads**															
	Truncated mean with maximized kappa from IGA; skilled overead of all positive images (n=196)	99		78		99		0.685 (0.786 in IGA sample)		2.5		5		84	
	Truncated mean with 90% skilled grader burden; with skilled overread of all positive images (n=115)	99		74		99		0.673 (0.741 in IGA sample)		2.4		6		90	

^a^N/A: not applicable.

^b^WHO: World Health Organization.

## Discussion

### Principal Findings

We developed and validated a decentralized, VRC using crowdsourcing to grade real-world smartphone-acquired images for the presence of trachoma. During the training of the VRC model, a “standalone” version of the VRC using only crowdsourcing was applied in a simulated skilled grader certification test (IGA) following WHO guidelines and was able to meet the minimum kappa agreement with an expert grader of >0.70. While we used the IGA guidelines originally developed for the Global Trachoma Mapping Project [24], which recommended a moderately high prevalence for the assessment (ie, 30-70%), more recent guidance has encouraged IGA with a minimum prevalence of 10% [26]. We see the greatest potential for applying telemedicine for trachoma grading in even lower prevalence environments and wanted to develop a tool that could produce prevalence estimates of ±2% with a TF prevalence of 5% or lower. Therefore, we tested the VRC using a data set with a low prevalence of 2%-3% for the training and test sets. In this environment, the most accurate VRC used crowdsourcing as a first pass, followed by skilled grading of positive images. This VRC model was able to maintain a kappa agreement of 0.775 with expert graders while generating a prevalence estimate of 2.70% (compared with the true prevalence of 2.87%).

### Trachoma Elimination

The current paradigm for clinical TF identification was developed in the milieu of high trachoma prevalence and prior to the era of global adoption of smartphone technology with wireless internet connectivity. Because the clinical sign of TF can be subtle, ongoing direct exposure to active disease is required for accurate field grading. In this sense, trachoma elimination programs can be conceived as the “victim of their own success” because as the disease is eliminated, it is harder to maintain a cadre of accurate field graders. While obviously a cause for celebration, the continued success of the enterprise is at risk without reevaluating and augmenting, or perhaps reinventing, acceptable district survey methods.

### Ophthalmic Photography

Ophthalmic photography has long been used for standardized diagnosis and monitoring for clinical and research purposes in many conditions, including trachoma [14-17,27]. The theoretical advantages of applying photography in TF elimination programs include decentralized grading (and thus avoiding bias), access to expert consultation for difficult cases, auditability of findings, and the ability to re-examine images in light of future evolution in our understanding of the disease. There is sufficient interest in the potential role of ophthalmic photography in trachoma elimination that the WHO convened 2 informal workshops on the topic in 2020 and committed to support the development of the evidence base for innovations in trachoma photography and imaging interpretation. A recent systematic review found 18 articles in the English language, peer-reviewed literature which, in aggregate, demonstrate that agreement between clinical assessment and photo-rendered grade is at least as good as the agreement between intergrader agreement among clinical grades [17]. Likewise, several groups have demonstrated the feasibility of smartphone photography for TF diagnosis [14,21], including the ability to upload and transmit images to a cloud server from rural field locations [21]. These results support a potential role for applying a telemedicine paradigm for district surveys, though the use of imaging in an elimination program at scale remains unproven.

An important component of a successful telemedicine program is the management and interpretation of imaging data. In a conventional clinical telemedicine program, images are generally interpreted in a reading center by a skilled grader with supervision by a licensed clinician [28]. Such a model can be robust and cost-efficient in clinical medicine but may not be scalable in large public health programs. We have therefore validated a VRC paradigm that incorporates crowdsourcing and skilled human grading to produce grades that would meet the standards set by the WHO for certification of a skilled grader.

### Use of Crowdsourcing

Crowdsourcing using unskilled internet users has been used to identify pathological features of diabetic retinopathy [19,20,29] and glaucoma [18,30] on fundus photographs as well as to classify surgical outcomes of ophthalmic surgery [31-33]. Aggregating the scores of a “crowd” of skilled graders has also been used in ophthalmology to generate robust consensus annotations of images for artificial intelligence (AI) algorithm training [34,35]. The advantages of crowdsourcing for image interpretation are that it capitalizes on human pattern recognition to identify features of the disease and ideally averages out individual biases (ie, over- or under-calling) to settle on an accurate classification. Using the AMT marketplace, large batches of >1000 images have been graded in parallel by hundreds of users in approximately 1 hour. We have shown that while this model is currently unable to provide a stand-alone classification for TF in a low prevalence environment; in a VRC model with skilled overreading of positive images, crowdsourcing accurately and efficiently identifies images free of TF. In the final validation model, only 205 of 1150 images identified as possible TF required a skilled grade.

### Limitations

The VRC model using crowdsourcing has some disadvantages. Because there is limited gatekeeping on the pool of workers, there is the potential for fraudulent users to contaminate the grading. We tried to minimize this by running the data set in 2 batches separated by several months in time and saw very similar usage characteristics in terms of the time spent per image and the amount of time taken to complete the entire batch. Ongoing quality control checks would need to be built into the system to ensure stability as in any diagnostic paradigm. In this study, we also examined individual worker characteristics and found that some users were clear outliers in category usage since they classified many more images with pathology than their peers. While removing these individuals’ 1160 grades improved our model, it was less stable and less efficient than simply truncating the highest and lowest grades. Specifically, by dropping 13.5% of the individual grades, only 478 of 1307 images had all 7 grades available, and 5 images had 3 or fewer grades (Figure S5 in Multimedia Appendix 1), in contrast with 1294 images with all grades available in the full training set. Methods to validate individual workers in real-time could be applied in future VRC models.

Perhaps, the biggest challenge to implementing telemedicine for TF is ensuring the data coming into the VRC system are of the highest possible quality. The data set used for this validation had 9% ungradable images [21], and while these were posted to AMT for crowdsourced assessment, gradability was not a required element of the task and there was limited instruction on what constituted an ungradable image. As such, workers were generally unable to identify expert-identified ungradable images as such (data not shown). Future refinements in photographic technique will likely improve the value of VRC grading.

### Conclusions and Future Directions

There is growing enthusiasm for automated classifiers built using AI methods to identify ophthalmic disease [36-39], culminating in the 2018 US FDA authorization of the first autonomous AI system in any field of medicine [40]. While we feel strongly that AI will likely contribute to or replace human grading for many visual tasks, we suggest for current use a VRC using crowdsourcing that can be flexible enough to incorporate AI as it matures. Furthermore, we recognize the realities of global priorities and funding timelines, with funding now set to expire in 2030, which is a short time to validate an autonomous AI system. We believe that a VRC system that meets or exceeds the standards set by the WHO and Tropical Data, and has value to add over current systems, could be deployed without delay to meet the needs of global elimination programs that are currently struggling to train and standardize skilled graders.

## Appendix

Multimedia Appendix 1 Supplementary figures.

Multimedia Appendix 2 Open data.

Multimedia Appendix 3 Data codebook.

## Abbreviations

AI: artificial intelligence
AMT: Amazon Mechanical Turk
AUROC: area under the receiver operating characteristic
CPT: current procedural terminology
FDA: Food and Drug Administration
ICAPS: Image Capture and Processing System
IGA: intergrader agreement test
ROC: receiver operating characteristic
TF: trachomatous inflammation—follicular
VRC: virtual reading center
WHO: World Health Organization

## Data Availability

All data generated or analyzed during this study are included in this published paper and in Multimedia Appendices 2 and 3.

## References

[1] Burton MJ, Faal HB, Ramke J, Ravilla T, Holland P, Wang N, West SK, Bourne RRA, Congdon NG, Foster A (2019). Announcing The Lancet Global Health Commission on global eye health. Lancet Global Health.

[2] Ferede AT, Dadi AF, Tariku A, Adane AA (2017). Prevalence and determinants of active trachoma among preschool-aged children in Dembia District, Northwest Ethiopia. Infect Dis Poverty.

[3] Flaxman SR, Bourne RRA, Resnikoff S, Ackland P, Braithwaite T, Cicinelli MV, Das A, Jonas JB, Keeffe J, Kempen JH, Leasher J, Limburg H, Naidoo K, Pesudovs K, Silvester A, Stevens GA, Tahhan N, Wong TY, Taylor HR, Vision Loss Expert Group of the Global Burden of Disease Study (2017). Global causes of blindness and distance vision impairment 1990-2020: a systematic review and meta-analysis. Lancet Glob Health.

[4] Lietman TM, Oldenburg CE, Keenan JD (2020). Trachoma: time to talk rradication. Ophthalmology.

[5] Williams LB, Prakalapakorn SG, Ansari Z, Goldhardt R (2020). Impact and trends in global ophthalmology. Curr Ophthalmol Rep.

[6] World Health Organization (2021). WHO Alliance for the Global Elimination of Trachoma by 2020: progress report on elimination of trachoma, 2020. Wkly Epidemiol Rec.

[7] WHO Alliance for the Global Elimination of Trachoma (1998). Report of the third meeting of the WHO Alliance for the Global Elimination of Trachoma. World Health Organization.

[8] West SK (2020). Milestones in the fight to eliminate trachoma. Ophthalmic Physiol Opt.

[9] (2019). Reaching the Last Mile Forum: Keynote Address. World Health Organization.

[10] Whitty CJM (2015). Political, social and technical risks in the last stages of disease eradication campaigns. Int Health.

[11] (2017). Network of WHO collaborating centres for trachoma: second meeting report. World Health Organization.

[12] Borlase A, Blumberg S, Callahan EK, Deiner MS, Nash SD, Porco TC, Solomon AW, Lietman TM, Prada JM, Hollingsworth TD (2021). Modelling trachoma post-2020: opportunities for mitigating the impact of COVID-19 and accelerating progress towards elimination. Trans R Soc Trop Med Hyg.

[13] (2021). Report of the 23rd meeting of the WHO alliance for the global elimination of trachoma by 2020. World Health Organization.

[14] Nesemann JM, Seider MI, Snyder BM, Maamari RN, Fletcher DA, Haile BA, Tadesse Z, Varnado NE, Cotter SY, Callahan EK, Emerson PM, Margolis TP, Lietman TM, Keenan JD (2020). Comparison of smartphone photography, single-lens reflex photography, and field-grading for trachoma. Am J Trop Med Hyg.

[15] Snyder BM, Sié A, Tapsoba C, Dah C, Ouermi L, Zakane SA, Keenan JD, Oldenburg CE (2019). Smartphone photography as a possible method of post-validation trachoma surveillance in resource-limited settings. Int Health.

[16] West SK, Taylor HR (1990). Reliability of photographs for grading trachoma in field studies. Br J Ophthalmol.

[17] Naufal F, West SK, Brady CJ (2022). Utility of photography for trachoma surveys: a systematic review. Surv Ophthalmol.

[18] Wang X, Mudie LI, Baskaran M, Cheng CY, Alward WL, Friedman DS, Brady CJ (2017). Crowdsourcing to evaluate fundus photographs for the presence of glaucoma. J Glaucoma.

[19] Brady CJ, Mudie LI, Wang X, Guallar E, Friedman DS (2017). Improving consensus scoring of crowdsourced data using the Rasch model: development and refinement of a diagnostic instrument. J Med Internet Res.

[20] Brady CJ, Villanti AC, Pearson JL, Kirchner TR, Gupta OP, Shah CP (2014). Rapid grading of fundus photographs for diabetic retinopathy using crowdsourcing. J Med Internet Res.

[21] Naufal F, Brady CJ, Wolle MA, Saheb Kashaf M, Mkocha H, Bradley C, Kabona G, Ngondi J, Massof RW, West SK (2021). Evaluation of photography using head-mounted display technology (ICAPS) for district trachoma surveys. PLoS Negl Trop Dis.

[22] Brady C, Bradley C, Wolle M, Mkocha H, Massof R, West S (2020). Virtual reading center can remotely identify follicular trachoma. Investig Ophthalmol Visual Sci.

[23] Solomon AW, Kello AB, Bangert M, West SK, Taylor HR, Tekeraoi R, Foster A (2020). The simplified trachoma grading system, amended. Bull World Health Organ.

[24] Solomon AW, Pavluck AL, Courtright P, Aboe A, Adamu L, Alemayehu W, Alemu M, Alexander NDE, Kello AB, Bero B, Brooker SJ, Chu BK, Dejene M, Emerson PM, Flueckiger RM, Gadisa S, Gass K, Gebre T, Habtamu Z, Harvey E, Haslam D, King JD, Mesurier RL, Lewallen S, Lietman TM, MacArthur C, Mariotti SP, Massey A, Mathieu E, Mekasha A, Millar T, Mpyet C, Muñoz BE, Ngondi J, Ogden S, Pearce J, Sarah V, Sisay A, Smith JL, Taylor HR, Thomson J, West SK, Willis R, Bush S, Haddad D, Foster A (2015). The global trachoma mapping project: methodology of a 34-country population-based study. Ophthalmic Epidemiol.

[25] Report of the third global scientific meeting on trachoma. World Health Organization.

[26] Courtright P, MacArthur C, Macleod C, Dejene M, Gass K, Harding-Esch E (2019). Tropical Data: Training System for Trachoma Prevalence Surveys.

[27] Solomon AW, Bowman RJC, Yorston D, Massae PA, Safari S, Savage B, Alexander NDE, Foster A, Mabey DCW (2006). Operational evaluation of the use of photographs for grading active trachoma. Am J Trop Med Hyg.

[28] Horton MB, Brady CJ, Cavallerano J, Abramoff M, Barker G, Chiang MF, Crockett CH, Garg S, Karth P, Liu Y, Newman CD, Rathi S, Sheth V, Silva P, Stebbins K, Zimmer-Galler I (2020). Practice guidelines for ocular telehealth-diabetic retinopathy, third edition. Telemed J E Health.

[29] Mitry D, Peto T, Hayat S, Morgan JE, Khaw K, Foster PJ (2013). Crowdsourcing as a novel technique for retinal fundus photography classification: analysis of images in the EPIC Norfolk cohort on behalf of the UK Biobank Eye and Vision Consortium. PLoS One.

[30] Mitry D, Peto T, Hayat S, Blows P, Morgan J, Khaw K, Foster PJ (2015). Crowdsourcing as a screening tool to detect clinical features of glaucomatous optic neuropathy from digital photography. PLoS One.

[31] Rootman DB, Bokman CL, Katsev B, Rafaelof M, Ip M, Manoukian N, Esfandiari M, Webb NM (2020). Crowdsourcing morphology assessments in oculoplastic surgery: reliability and validity of lay people relative to professional image analysts and experts. Ophthalmic Plast Reconstr Surg.

[32] Paley GL, Grove R, Sekhar TC, Pruett J, Stock MV, Pira TN, Shields SM, Waxman EL, Wilson BS, Gordon MO, Culican SM (2021). Crowdsourced assessment of surgical skill proficiency in cataract surgery. J Surg Educ.

[33] Kim TS, O'Brien M, Zafar S, Hager GD, Sikder S, Vedula SS (2019). Objective assessment of intraoperative technical skill in capsulorhexis using videos of cataract surgery. Int J Comput Assist Radiol Surg.

[34] Schaekermann M, Hammel N, Terry M, Ali TK, Liu Y, Basham B, Campana B, Chen W, Ji X, Krause J, Corrado GS, Peng L, Webster DR, Law E, Sayres R (2019). Remote tool-based adjudication for grading diabetic retinopathy. Transl Vis Sci Technol.

[35] Gulshan V, Peng L, Coram M, Stumpe MC, Wu D, Narayanaswamy A, Venugopalan S, Widner K, Madams T, Cuadros J, Kim R, Raman R, Nelson PC, Mega JL, Webster DR (2016). Development and validation of a deep learning algorithm for detection of diabetic retinopathy in retinal fundus photographs. JAMA.

[36] Kim MC, Okada K, Ryner AM, Amza A, Tadesse Z, Cotter SY, Gaynor BD, Keenan JD, Lietman TM, Porco TC (2019). Sensitivity and specificity of computer vision classification of eyelid photographs for programmatic trachoma assessment. PLoS One.

[37] Campbell JP, Mathenge C, Cherwek H, Balaskas K, Pasquale LR, Keane PA, Chiang MF, American Academy of Ophthalmology Task Force on Artificial Intelligence (2021). Artificial intelligence to reduce ocular health disparities: moving from concept to implementation. Transl Vis Sci Technol.

[38] Abràmoff MD, Leng T, Ting DSW, Rhee K, Horton MB, Brady CJ, Chiang MF (2020). Automated and computer-assisted detection, classification, and diagnosis of diabetic retinopathy. Telemed J E Health.

[39] Socia D, Brady CJ, West SK, Cockrell RC (2022). Detection of trachoma using machine learning approaches. PLoS Negl Trop Dis.

[40] Abràmoff MD, Lavin PT, Birch M, Shah N, Folk JC (2018). Pivotal trial of an autonomous AI-based diagnostic system for detection of diabetic retinopathy in primary care offices. NPJ Digit Med.

