# Supplementary material for: A Virtual Reading Center Model Using Crowdsourcing to Grade Photographs for Trachoma: Validation Study
Source: J Med Internet Res. 2023 Apr 6;25:e41233. doi: 10.2196/41233 (PMC10132003; doi:10.2196/41233)

**Multimedia Appendix 1. Supplementary figures.**

Figure S1. Example everted upper eyelid photograph from the experimental data set without trachomatous inflammation—follicular.


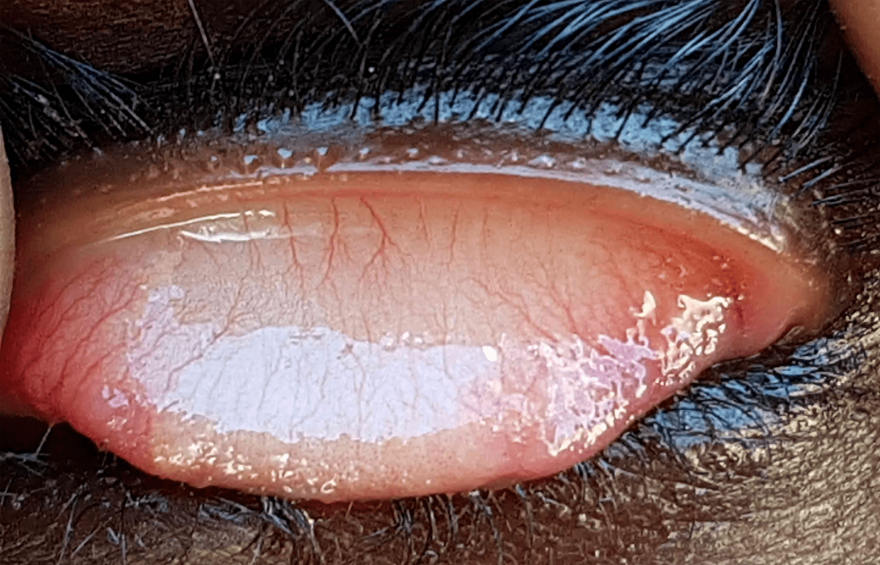


Figure S2. Example everted upper eyelid photograph from the experimental data set with trachomatous inflammation—follicular.


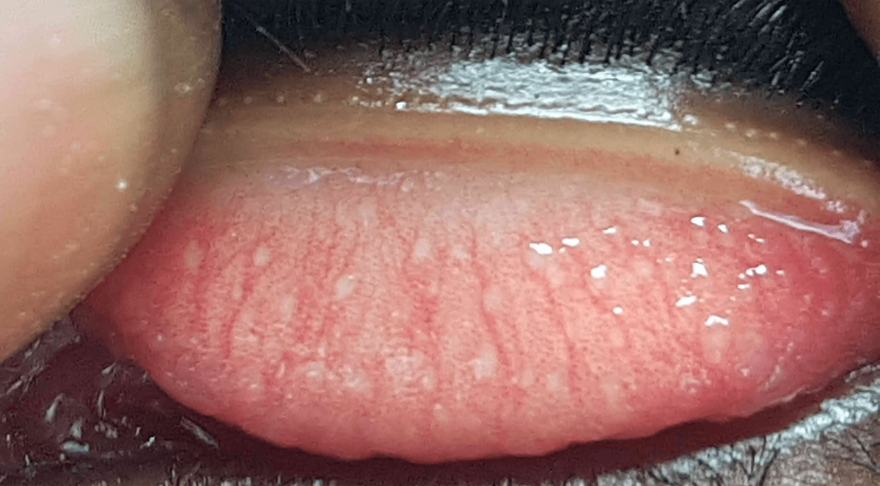


Figure S3. Distribution of the number of tasks completed by each unique Amazon Mechanical Turk user.


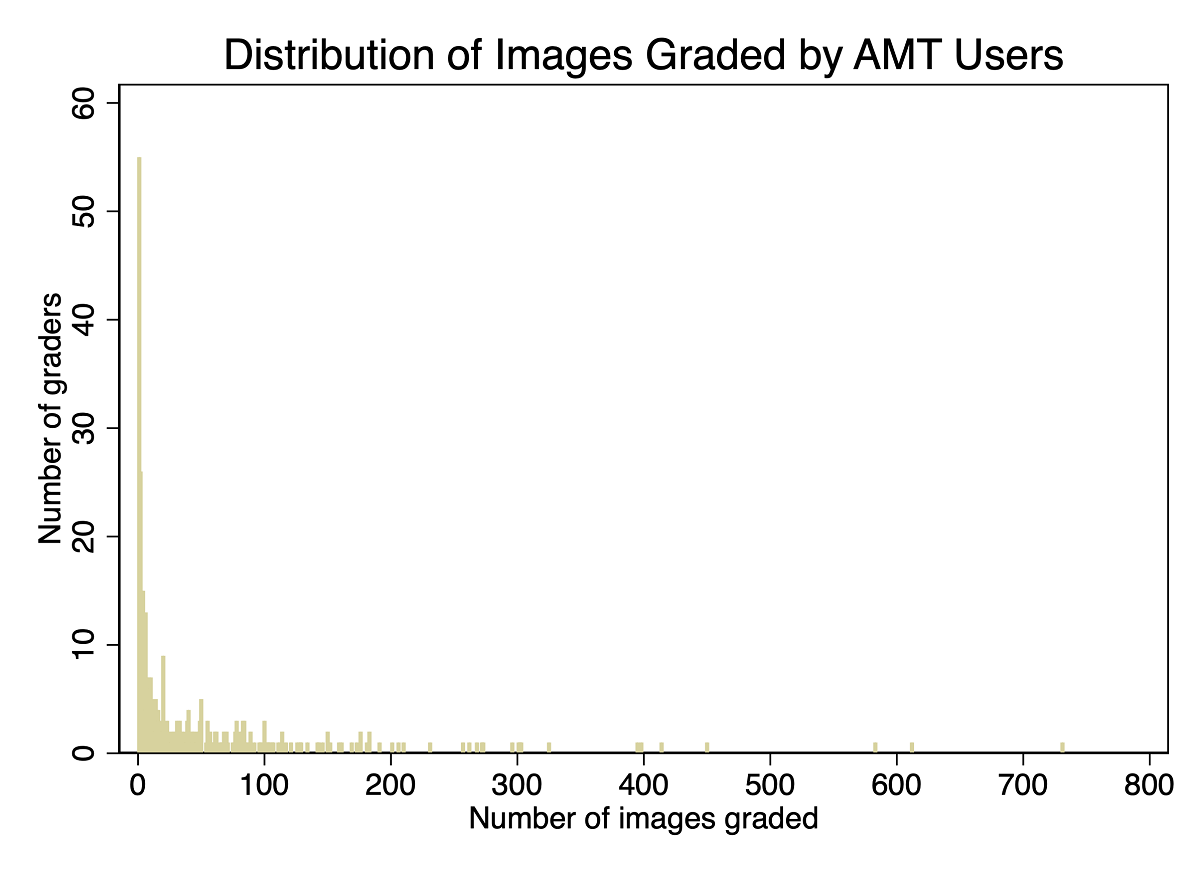


Figure S4. Distribution of crowdsourced raw scores of all images in the dataset. Each score was the sum of 7 independent grades ranging from 0= “Not TF” to 3= “Definitely TF.” The distribution is right-skewed reflecting the low prevalence of disease in this sample.


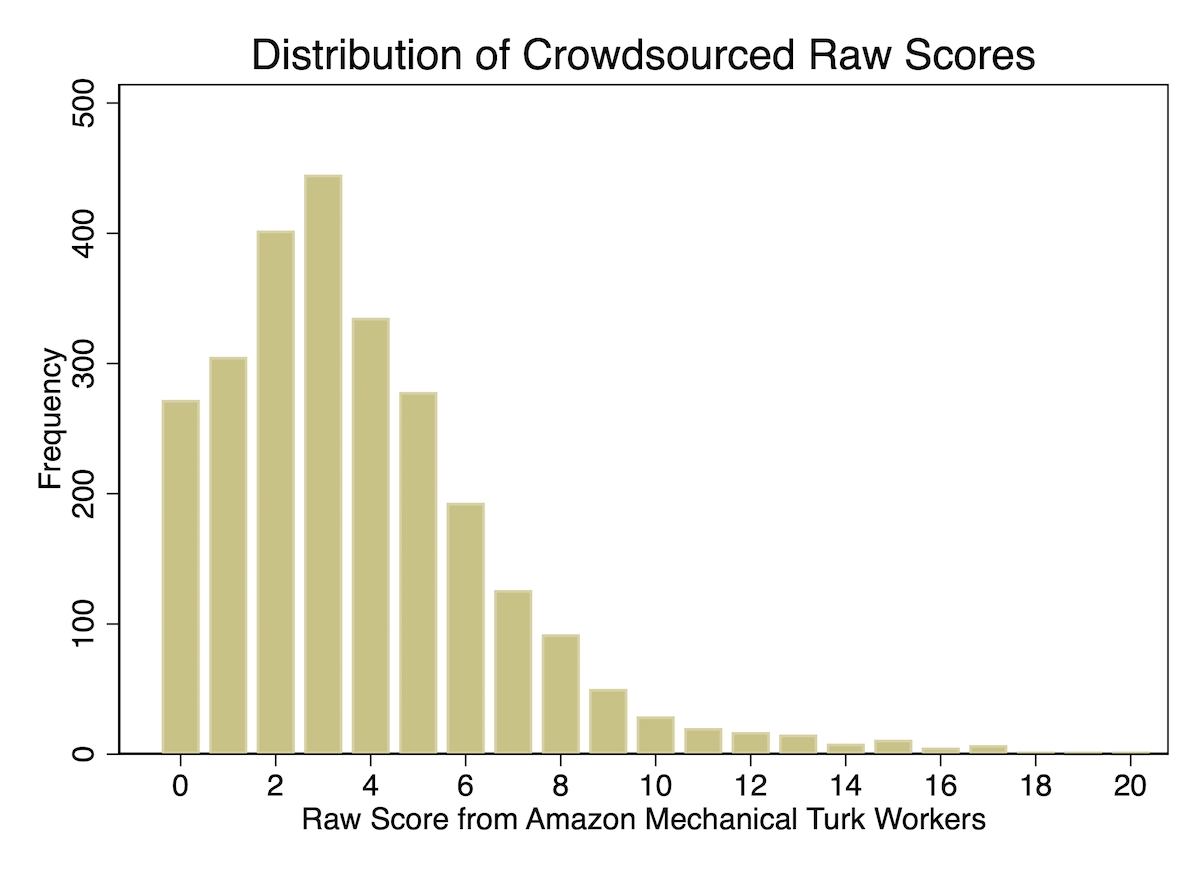


Figure S5. Uneven distribution of the number of grades available for analysis after removing “variable graders” led to concern for instability of image raw scores. This supported our decision to truncated the highest and lowest score for each image, which was more analytically efficient and affected all images equally.


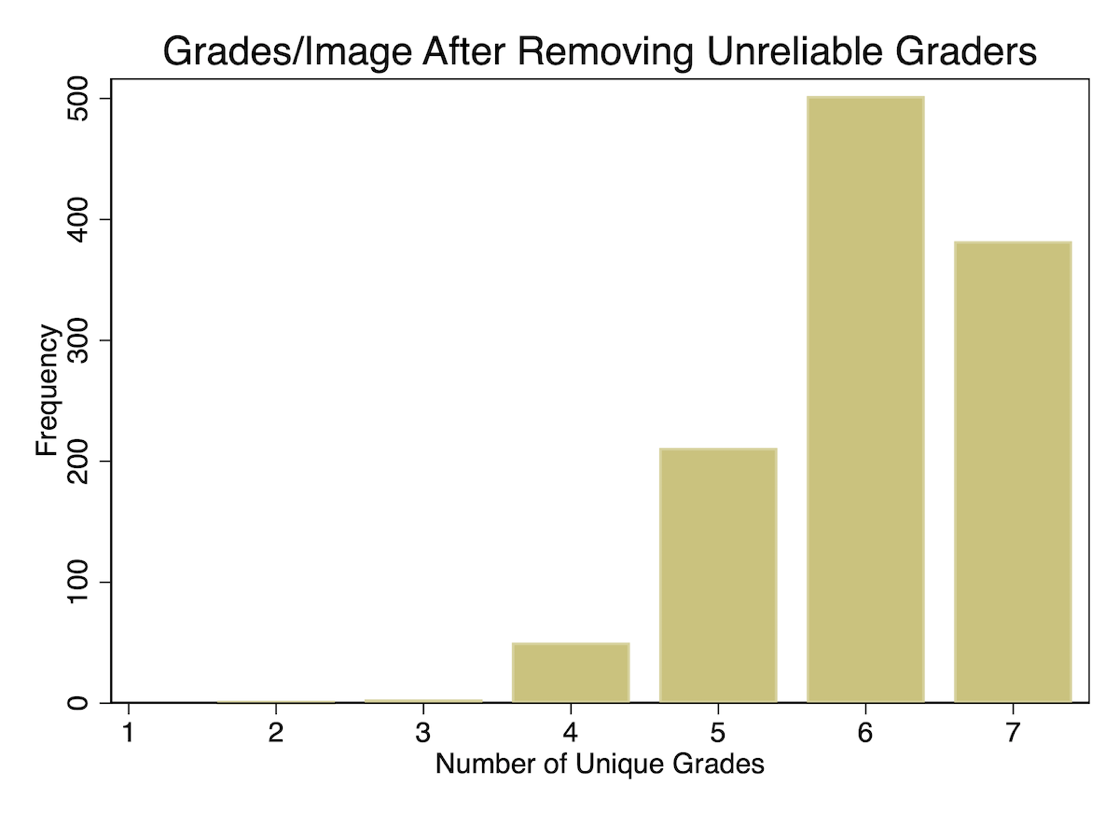

Supplement: Multimedia Appendix 1 [file jmir_v25i1e41233_app1.docx]
